# ACCEPTABILITY AND FEASIBILITY OF IMPLEMENTING THE DEMENTIA CARE PRACTICE RECOMMENDATIONS IN CARE COMMUNITIES

**DOI:** 10.1093/geroni/igae098.3068

**Published:** 2024-12-31

**Authors:** Boeun Kim, Basia Belza, Lorna Prophater, Emily Waddington, Shawn Johnson, Sam Fazio

**Affiliations:** University of Iowa, Iowa, United States; University of Washington, Seattle, Washington, United States; Alzheimer’s Association, Chicago, Illinois, United States; Alzheimer’s Association, Chicago, Illinois, United States; The Alzheimer’s Association, Chicago, Illinois, United States; Alzheimer’s Association, Chicago, Illinois, United States

## Abstract

With growing numbers of residents living with dementia in care communities, providing quality care is challenging. Alzheimer’s Association developed an intervention to increase the adoption of person-centered care in care communities using a coaching approach based on the Dementia Care Practice Recommendations (DCPR). A cluster randomized-controlled study has been implemented to evaluate the intervention since December 2022. This study aims to report interim findings. Care communities in an intervention group met with a coach monthly over 6 months to assess the level of adoption of DCPR and increase and maintain person-centered care practices. Care communities in a control group received monthly emails not relevant to person-centered care. Employee satisfaction (job satisfaction, team building and communication, scheduling and staffing, training, management and leadership), person-centered practices (workplace practices, individualized care and services, caregiver-resident relationships), and confidence in dementia scale were assessed pre-intervention (time1), post-intervention (time2), and 3-month follow-up after completion of the intervention (time3). As of February 2024, 292 care providers from 53 care communities enrolled, 164 care providers from 35 care communities completed the 6-month intervention, and 113 care providers from 25 care communities filled out 3-month follow-up assessments. We found that scheduling and staffing satisfaction (time3), management and leadership satisfaction (time3), overall employee satisfaction (time3), and person-centered practices in individualized care and services (time2 and 3) were significantly higher in intervention groups than control groups according to generalized estimating equation estimates. This study provides preliminary evidence of the efficacy of the DCPR intervention in promoting employee satisfaction and person-centered practices.

